# Efficacy and safety of perampanel monotherapy in patients with focal‐onset seizures with newly diagnosed epilepsy or recurrence of epilepsy after a period of remission: The open‐label Study 342 (FREEDOM Study)

**DOI:** 10.1002/epi4.12398

**Published:** 2020-06-07

**Authors:** Takamichi Yamamoto, Sung Chul Lim, Hirotomo Ninomiya, Yuichi Kubota, Won Chul Shin, Dong Wook Kim, Dong Jin Shin, Tohru Hoshida, Koji Iida, Taku Ochiai, Risa Matsunaga, Hiroyuki Higashiyama, Hidetaka Hiramatsu, Ji Hyun Kim

**Affiliations:** ^1^ Seirei Hamamatsu General Hospital Hamamatsu Japan; ^2^ The Catholic University of Korea St. Vincent Hospital Gyeonggi‐do Republic of Korea; ^3^ Itami City Hospital Hyogo Japan; ^4^ TMG Asaka Medical Center Saitama Japan; ^5^ Kyung Hee University Hospital at Gangdong Seoul Republic of Korea; ^6^ Konkuk University School of Medicine Seoul Republic of Korea; ^7^ Gachon University Gil Medical Center Incheon Republic of Korea; ^8^ National Hospital Organization Nara Medical Center Nara Japan; ^9^ Hiroshima University Hiroshima Japan; ^10^ Ochiai Neurological Clinic Saitama Japan; ^11^ Eisai Co., Ltd. Tokyo Japan; ^12^ Formerly: Eisai Co., Ltd. Tokyo Japan; ^13^ Korea University Guro Hospital Seoul Republic of Korea; ^14^ Present address: Tokyo Women’s Medical University Medical Center East Tokyo Japan; ^15^ Present address: Takanohara Central Hospital Nara Japan

**Keywords:** antiepileptic drug, focal‐onset seizures, monotherapy, perampanel, seizure freedom

## Abstract

**Objective:**

Our study assessed perampanel monotherapy in patients (aged ≥12 years) with focal‐onset seizures (FOS) with or without focal to bilateral tonic‐clonic seizures (FBTCS) in Japan and South Korea.

**Methods:**

Study 342 (NCT03201900; FREEDOM) is a single‐arm, open‐label, Phase III study. Patients initially received perampanel in a 32‐week 4‐mg/d Treatment Phase (6‐week Titration; 26‐week Maintenance Periods). If they experienced a seizure during the 4‐mg/d Maintenance Period, they could be up‐titrated to 8 mg/d across an additional 30‐week Treatment Phase (4‐week Titration; 26‐week Maintenance Periods). Primary endpoint was the seizure‐freedom rate during the Maintenance Period (4 mg/d and last evaluated dose [4 or 8 mg/d]). Secondary endpoints included time to first seizure onset and to withdrawal during Maintenance. Treatment‐emergent adverse events (TEAEs) were monitored.

**Results:**

At data cutoff (February 28, 2019), 89 patients with FOS (84 [94.4%] with newly diagnosed epilepsy and 5 [5.6%] with recurrence of epilepsy after a period of remission) had received ≥1 perampanel dose; 16 patients discontinued during the 4‐mg/d Titration Period, meaning 73 patients entered the 4‐mg/d Maintenance Period and were included in the primary analysis set for efficacy. Seizure‐freedom rate in the 26‐week Maintenance Period was 46/73 (63.0%; 95% confidence interval [CI]: 50.9‐74.0) at 4 mg/d and 54/73 (74.0%; 95% CI: 62.4‐83.5) at 4 or 8 mg/d. Cumulative probability of seizure‐onset and withdrawal rates during Maintenance was 30.8% (95% CI: 21.5‐43.0) and 23.7% (95% CI: 15.4‐35.3) at 4 mg/d, and 18.2% (95% CI: 11.0‐29.3) and 23.3% (95% CI: 15.2‐34.8) at 4 or 8 mg/d. Perampanel was generally well tolerated, and the most common TEAE was dizziness.

**Significance:**

Perampanel monotherapy (4 to 8 mg/d) was efficacious and consistent with the known safety profile up to 26 weeks in patients (≥12 years) with primarily newly diagnosed FOS with or without FBTCS.

**Abbreviations**
AED: antiepileptic drug
CI: confidence interval
ECG: electrocardiogram
EEG: electroencephalogram
FAS: focal aware seizures
FBTCS: focal to bilateral tonic‐clonic seizures
FDA: Food and Drug Administration
FIAS: focal impaired awareness seizures
FOS: focal‐onset seizures
ICH: International Conference on Harmonization
ILAE: International League Against Epilepsy
ITT: intent‐to‐treat
MedDRA: Medical Dictionary for Regulatory Activities
mITT: modified intent‐to‐treat
QD: once daily
SAS: Safety Analysis Set
SD: standard deviation
TEAE: treatment‐emergent adverse event

**Key Points**

- Perampanel monotherapy (4 mg/d, or titrated to 8 mg/d after a seizure) was studied in primarily newly diagnosed focal‐onset seizures
- Seizure‐freedom rate in the 26‐week Maintenance Period was 46/73 (63.0%) at 4 mg/d and 54/73 (74.0%) at last evaluated dose (4 or 8 mg/d)
- From other monotherapy studies with other AEDs, the seizure‐freedom rate fulfilled prespecified efficacy criteria
- The safety profile was consistent with Phase III studies of adjunctive perampanel
- Perampanel monotherapy (4 to 8 mg/d) may be suitable for currently untreated focal‐onset seizures with/without FBTCS

## 1 INTRODUCTION

It has been shown that some patients with newly diagnosed epilepsy can achieve seizure freedom following treatment with antiepileptic drug (AED) monotherapy while other patients are not able to achieve seizure freedom to the same extent.^1^, ^2^, ^3^, ^4^, ^5^, ^6^ The likelihood of achieving seizure freedom reduces with the number of AEDs, with patients who have received 2 successive AEDs having a significantly lower seizure‐freedom rate than patients treated with 1 AED.^6^ Guidelines provided by the International League Against Epilepsy (ILAE) offer specific recommendations regarding the use of AEDs as monotherapy.^7^

Perampanel is an orally active, noncompetitive, selective α‐amino‐3‐hydroxy‐5‐methyl‐4‐isoxazolepropionic acid receptor antagonist.^8^ In the United States, perampanel is approved as adjunctive therapy and monotherapy for the treatment of focal‐onset seizures (FOS; previously known as partial‐onset seizures) with or without focal to bilateral tonic‐clonic seizures (FBTCS; previously known as secondarily generalized seizures) in patients 4 years of age and above, and as adjunctive treatment of generalized‐onset tonic‐clonic seizures (previously known as primary generalized tonic‐clonic seizures) in patients 12 years of age and above.^9^ For FOS, the recommended maintenance dose range of perampanel is 8 mg to 12 mg once daily. Some may respond to a dose of 4 mg/d; however, the efficacy of this dose has not been fully clarified. Approval of perampanel as monotherapy for FOS was based on a Food and Drug Administration (FDA) policy that allows extrapolation of efficacy and safety data from adjunctive AED studies to the monotherapy setting.^10^ Although the efficacy and safety of perampanel monotherapy have been demonstrated in patients with refractory epilepsy,^11^, ^12^ limited information is available, and the efficacy and safety of perampanel administered as monotherapy are yet to be thoroughly investigated in patients with newly diagnosed epilepsy.^13^

Study 342 is an open‐label, single‐arm, Phase III clinical trial and the first study of perampanel monotherapy in patients with newly diagnosed FOS (with or without FBTCS) in Japan and South Korea. This study is without a control arm due to ethical concerns regarding giving placebo to untreated patients with epilepsy.^14^ The main purpose of this study is to evaluate the seizure‐freedom rate during the 26‐week Maintenance Period in patients with FOS with newly diagnosed epilepsy or recurrence of epilepsy after a period of remission. This study consists of 4 phases: a Pretreatment Phase, Treatment Phase, Extension Phase, and Follow‐up Phase. The initial Treatment Phase was the 4‐mg/d Treatment Phase (Titration Period and 4‐mg/d Maintenance Period). If patients experienced seizures during the 4‐mg/d Maintenance Period, the investigators judged their transition to the 8‐mg/d Treatment Phase (Titration Period and 8‐mg/d Maintenance Period). In this paper, we describe efficacy and safety data from the Treatment Phase of Study 342.

## 2 METHODS

### 2.1 Study design

**FIGURE 1:**
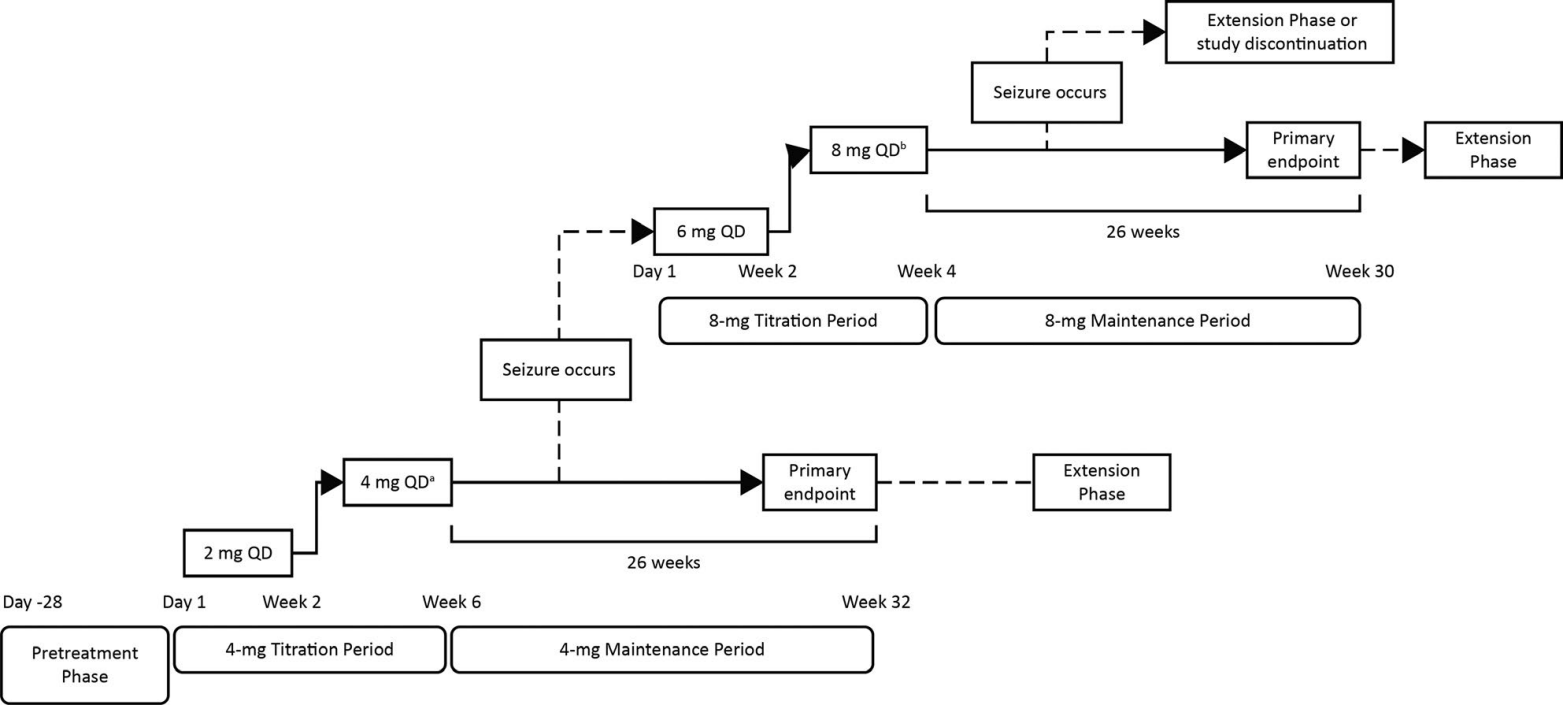
Study design. ^a^In the event of tolerability issues, the dose of perampanel could be reduced from 4 mg/d to 2 mg/d during Weeks 3 and 4 of the Titration Period, at the investigators’ discretion. If the dose could not be up‐titrated back to 4 mg/d, patients were discontinued from the study. ^b^Patients experiencing seizures while receiving perampanel 4 mg/d could receive perampanel 8 mg/d at the investigators’ discretion. If the 8‐mg/d dose was not tolerated, patients could be down‐titrated to 6 mg/d and continue the Maintenance Period. If patients experienced seizures while receiving perampanel 6 or 8 mg/d, or if the 6‐mg/d dose was not tolerated, they ended the Treatment Phase. Abbreviation: QD, once daily

Study 342 (ClinicalTrials.gov Identifier: NCT03201900; FREEDOM) is an uncontrolled, single‐arm, open‐label, Phase III study conducted at 31 sites in Japan and 7 sites in South Korea. During the Pretreatment Phase (maximally 4 weeks), patients were screened and assessed for their eligibility to participate in the study. Screening occurred between Day −28 and Day 1 (Day 1 is the date of first dose of perampanel 2 mg) (Figure 1). Patients who completed screening and met the inclusion/exclusion criteria began the 32‐week 4‐mg/d Treatment Phase (6‐week Titration Period; 26‐week Maintenance Period).

During the Titration Period, patients were initiated on once‐daily oral perampanel 2 mg/d before bedtime for 2 weeks (Weeks 1 and 2) and, if no tolerability issues arose, up‐titrated to 4 mg/d for 4 weeks (Weeks 3 through 6). During Weeks 3 and 4, down‐titration of perampanel dose to 2 mg/d was allowed at the investigators’ discretion in patients who experienced tolerability issues (in which case, patients were to visit the investigational site within 4 weeks of starting treatment and perampanel dose would be up‐titrated to 4 mg/d). During Weeks 5 and 6, all patients continued to receive perampanel 4 mg/d. Patients who tolerated perampanel 4 mg/d at the end of the Titration Period would continue with treatment in the 4‐mg/d Maintenance Period. Patients who could not comply with this schedule or could not tolerate perampanel treatment at the end of the Titration Period were discontinued from the study. Patients who experienced seizures during the 4‐mg/d Maintenance Period ended this period and underwent transition, based on the investigators’ assessment of safety and tolerability, to a 4‐week Titration Period (6 mg/d for 2 weeks, then 8 mg/d for 2 weeks) before entering into a 26‐week 8‐mg/d Maintenance Period. If patients could not tolerate administration of perampanel at 8 mg/d, down‐titration of perampanel dosing to 6 mg/d was allowed at the investigators’ discretion. After down‐titration, patients were allowed to continue the study at a dose of 6 mg/d. Patients who experienced seizures or who could not continue dosing at least 6 mg/d due to tolerability issues ended the Treatment Phase.

After completion of the Treatment Phase, patients who agreed would enter the Extension Phase to continue receiving perampanel monotherapy at their last dose achieved at the end of the Maintenance Period. Patients who finished or discontinued the study returned for the Follow‐up Phase, which occurred 4 weeks after the withdrawal of perampanel.

### 2.2 Patients

Eligible patients were 12‐74 years of age with a diagnosis of FOS (with or without FBTCS) according to the 2017 ILAE Classification of Epileptic Seizures (the 1981 classification was referred to in the study protocol). Diagnosis was established by clinical history and an electroencephalogram (EEG), which were consistent with FOS. Patients with a normal EEG could be included provided they met the other diagnosis criteria according to clinical history.

All patients had newly diagnosed epilepsy or recurrence of epilepsy after a period of remission (patients with recurrence must have relapsed at least 2 years after the end of their last AED treatment). In addition, patients should have experienced at least 2 unprovoked seizures, separated by ≥24 hours, within a year prior to the Pretreatment Phase, of which at least 1 unprovoked seizure (but <20 seizures) must have occurred in the 12 weeks prior to the Pretreatment Phase. Key exclusion criteria were patients who only experienced focal aware seizures (FAS; previously known as simple partial seizures) without motor signs; had received prior AEDs (except for those used as rescue treatment) within 12 weeks prior to the Pretreatment Phase of the study or for more than 2 weeks in total within 2 years prior to the Pretreatment Phase of the study; had any history of AED polytherapy; or had seizures caused by progressive central nervous system abnormality detected via computed tomography or magnetic resonance imaging within 1 year prior to the Pretreatment Phase of the study. Furthermore, use of concomitant medications including antipsychotic drugs, cytochrome P450‐inducing foods and medications, and other AEDs was not permitted unless emergency care was needed due to the patient experiencing status epilepticus, uncontrolled seizures, or clusters of seizures.

### 2.3 Efficacy and safety endpoints

Efficacy endpoints were based on a seizure diary filled out by a patient or a parent/caregiver. The primary endpoint was the seizure‐freedom rate in the 26‐week Maintenance Period for patients with FOS (defined as the number [percentage] of patients with FOS who achieved seizure freedom). The number (percentage) of patients with FOS who achieved seizure freedom during the 26‐week Maintenance Period of 4 mg/d and regardless of perampanel dose (last evaluated dose of 4 or 8 mg/d) was calculated. The secondary endpoints were time to first seizure onset (defined as the period from the first perampanel dose in the Maintenance Period to first seizure onset) and time to withdrawal from the study (defined as the period from the first dose of perampanel in the Maintenance Period to the date of study withdrawal). Seizure‐freedom rate by FOS type (FBTCS, focal impaired awareness seizures [FIAS; previously known as complex partial seizures], and FIAS and/or FBTCS) was an exploratory endpoint.

Treatment‐emergent adverse events (TEAEs) were recorded and defined as: adverse events (AEs) that emerged from the first perampanel dose to the last visit or 28 days after the patient's last dose (whichever came later), having been absent at their Baseline visit or reemerged during treatment, having been present at their Baseline visit but stopped before treatment, or AEs which worsened in severity during treatment relative to the Baseline visit (if the AE was continuous). TEAEs were recorded using the Medical Dictionary for Regulatory Activities Version 21.0, from the time of patient consent through the last visit; serious TEAEs were recorded for 28 days after the last dose. Clinical laboratory parameters, vital signs, weight, and 12‐lead electrocardiogram (ECG) were assessed during the Pretreatment Phase, Days 1, 43, 141, 225, and the Follow‐up Phase.

### 2.4 Statistical analyses

The Intent‐to‐Treat (ITT) Analysis Set was defined as all patients who provided informed consent, received at least 1 dose of perampanel, and had at least 1 post‐dose primary efficacy measurement. Efficacy endpoints were assessed in the modified ITT (mITT) Analysis Set (defined as patients from the ITT Analysis Set who entered the perampanel 4‐mg/d Maintenance Period and had at least 1 post‐dose primary efficacy measurement in the Maintenance Period). A sensitivity analysis for the primary efficacy endpoint (seizure‐freedom rate during the 4‐mg/d Maintenance Period) was performed on the ITT Analysis Set.

In this study, the efficacy of perampanel was confirmed if the lower 95% confidence interval (CI), calculated using Clopper‐Pearson's exact method, for the seizure‐freedom rate during the Maintenance Period was >40%. This prespecified threshold was determined based on the expected seizure‐freedom rate of other AEDs from historical AED monotherapy studies (50%).^15^, ^16^, ^17^, ^18^, ^19^ In addition, the 2013 ILAE evidence review of AED efficacy and effectiveness as initial monotherapy for epileptic seizures and syndromes set 50% as an absolute minimum point estimate for efficacy of an adequate comparator and thus a lower boundary of 40% for noninferiority comparisons; a relative difference of >20% versus the adequate comparator's efficacy point estimate was considered to have a noninferior margin if its 95% lower CI limit was above this lower acceptable cutoff.^7^

Median time to first seizure onset and time to withdrawal from the study were estimated using the Kaplan‐Meier method. Patients who withdrew from the study before they experienced a seizure were censored at the time of study discontinuation.

The Safety Analysis Set (SAS) was defined as all patients who provided informed consent, had received at least 1 dose of perampanel, and had at least 1 post‐dose safety assessment. All safety analyses were performed based on the SAS and summarized using descriptive statistics.

All statistical analyses were performed using Statistical Analysis System software version 9.2 or higher.

### 2.5 Standard protocol approvals, registration, and patient consent

Study 342 was conducted in accordance with the Declaration of Helsinki, and the Committee for Proprietary Medicinal Products and International Conference on Harmonization (ICH)‐E6 Guideline for Good Clinical Practice CPMP/ICH/135/95. The study protocol, amendments, and informed consent form were reviewed by independent ethics committees or institutional review boards before the study was initiated.

Before study participation, investigators obtained written informed consent from each patient or assent from each young patient, followed by the consent of their parent/guardian. Verbal consent was obtained from patients who were unable to provide written consent.

## 3 RESULTS

### 3.1 Patients

**FIGURE 2:**
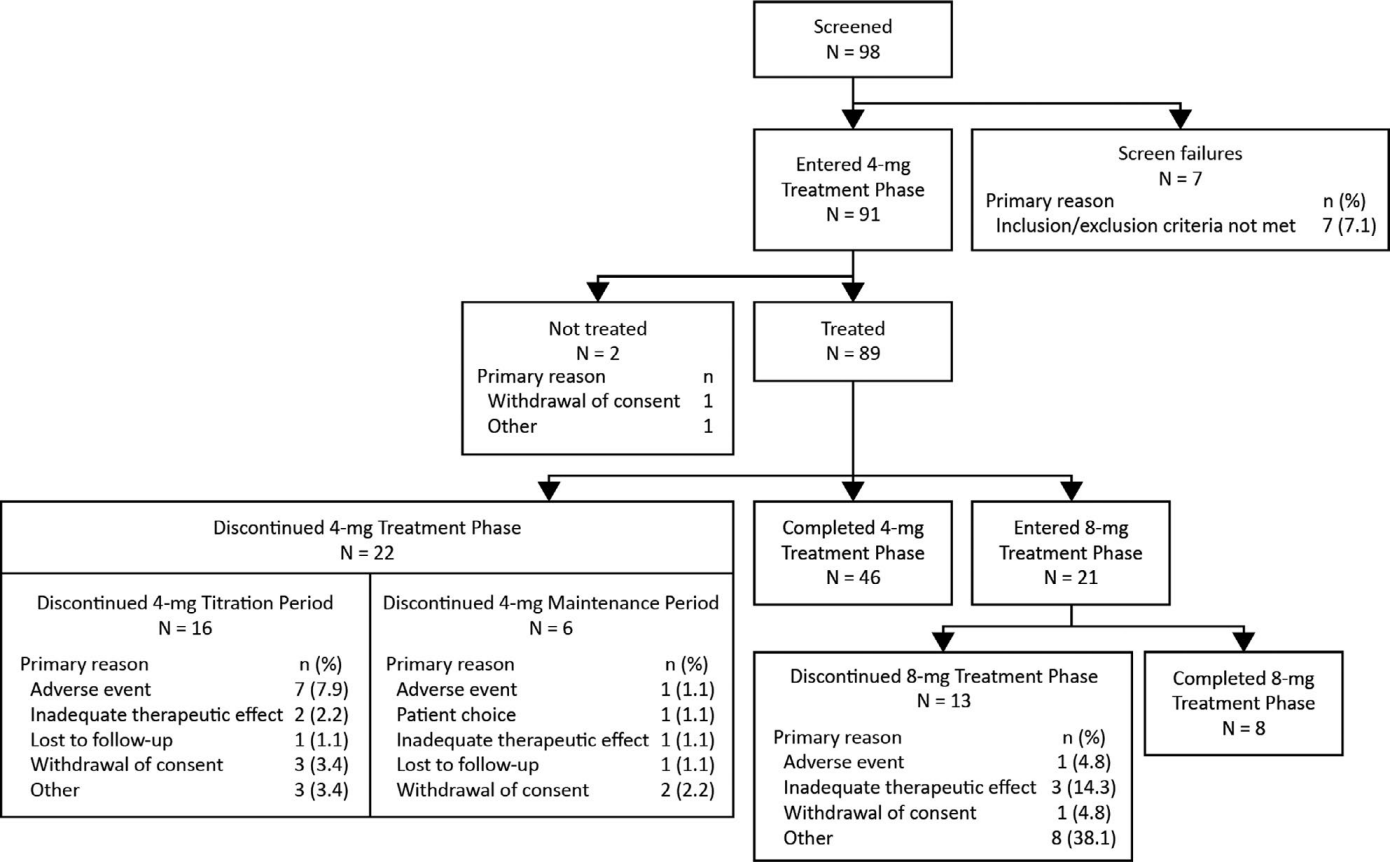
Patient disposition

Patients were enrolled between June 2017 and February 2018, and the data cutoff for the current analysis was February 28, 2019. Patient disposition is presented in Figure 2. A total of 91 patients entered the Treatment Phase; 89 patients received at least 1 dose of perampanel and were included in both the ITT and SAS populations (Japan, n = 43; South Korea, n = 46). Of these 89 patients, 46 (51.7%) patients completed the 4‐mg/d Treatment Phase of perampanel, and 22 (24.7%) patients discontinued from the 4‐mg/d Treatment Phase (16 during the 4‐mg/d Titration Period and 6 during the 4‐mg/d Maintenance Period). The most common reasons for discontinuation from the 4‐mg/d Treatment Phase were due to AEs (n = 8; 9.0%), withdrawal of consent (n = 5; 5.6%), and inadequate therapeutic effect (n = 3; 3.4%). Seventy‐three patients who entered the 4‐mg/d Maintenance Period and had at least 1 post‐dose primary efficacy measurement were included in the mITT Analysis Set (the 16 patients who discontinued during the 4‐mg/d Titration Period were excluded: 7 due to AEs, 3 withdrawal of consent, 2 inadequate therapeutic effect, 1 lost to follow‐up, and 3 other reasons). The remaining 21 (23.6%) patients entered the perampanel 8‐mg/d Treatment Phase due to the occurrence of seizures during the 4‐mg/d Maintenance Period.

Patient characteristics at baseline are presented in Table 1. Eighty‐four (94.4%) patients had newly diagnosed epilepsy, and 5 (5.6%) patients had recurrence of epilepsy after a period of remission (prior to this study, these patients were diagnosed with FIAS [2 patients], FBTCS [2 patients], or unknown seizure type [1 patient]). There were 57 (64.0%) patients who had FBTCS, 54 (60.7%) patients who had FIAS, and 10 (11.2%) patients who had FAS with motor signs. Median baseline (minimum, maximum) seizure frequency per 12 weeks in the mITT Analysis Set was 2.0 (0.9, 21.2).

**TABLE 1:** Baseline patient characteristics (Safety Analysis Set)

	Perampanel (N = 89)
Mean age, years (SD)	42.1 (18.2)
Age group, n (%)	
<18 y	7 (7.9)
18 to <65 y	71 (79.8)
≥65 y	11 (12.4)
Female, n (%)	44 (49.4)
Country, n (%)	
Japan	43 (48.3)
South Korea	46 (51.7)^a^
History of epilepsy, n (%)	
Newly diagnosed	84 (94.4)
Recurrence after a period of remission	5 (5.6)
Mean time since latest diagnosis of epilepsy, months (SD)^b^	2.1 (12.8)
Epileptic syndrome, n (%)^c^	
Symptomatic	40 (44.9)
Cryptogenic	39 (43.8)
Idiopathic	4 (4.5)
Unknown	6 (6.7)
Seizure type, n (%)^d^	
FBTCS	57 (64.0)
FIAS	54 (60.7)
FAS with motor signs	10 (11.2)
FAS without motor signs^e^	4 (4.5)
Etiology, n (%)^f^	
Head injury/cranial trauma	2 (2.2)
CNS infection(s)	1 (1.1)
Stroke	2 (2.2)
Structural brain anomalies or malformations	11 (12.4)
Vascular brain anomalies	1 (1.1)
Sleep disorder(s)	0
Other	3 (3.4)
Unknown	69 (77.5)
Suspected localization of epileptogenic region, n (%)^g^	
Temporal lobe	51 (57.3)
Frontal lobe	29 (32.6)
Parietal lobe	5 (5.6)
Occipital lobe	2 (2.2)
Other	0
Unknown	11 (12.4)

Abbreviations: CNS, central nervous system; FAS, focal aware seizures; FBTCS, focal to bilateral tonic‐clonic seizures; FIAS, focal impaired awareness seizures; SD, standard deviation.

^a^ Includes 1 patient of Chinese origin and 1 patient of “other Asian” origin.

^b^ Defined as the (screening date – date of diagnosis + 1)/30.5, rounded up to 1 decimal place.

^c^ Only a patient's primary epileptic syndrome is listed.

^d^ Multiple seizure types may have been recorded.

^e^ These patients also had other seizure types (patients with only FAS without motor signs were excluded from the study): 1 patient also presented with FIAS, and FBTCS; 1 patient also had FBTCS; 1 patient also had FIAS; and 1 patient also had FAS with motor signs, and FIAS.

^f^ Only a patient's primary reason is listed.

^g^ Multiple suspected localizations of the epileptogenic region may have been recorded.

### 3.2 Efficacy outcomes

In relation to the primary efficacy endpoint in the mITT Analysis Set, the seizure‐freedom rate in the Maintenance Period for all FOS was 46/73 (63.0%; 95% CI: 50.9‐74.0) at 4 mg/d and 54/73 (74.0%; 95% CI: 62.4‐83.5) at 4 or 8 mg/d (Table 2). The sensitivity analysis for the ITT Analysis Set resulted in a seizure‐freedom rate in the Maintenance Period of 46/89 (51.7%; 95% CI 40.8‐62.4) at 4 mg/d.

**TABLE 2:** Efficacy analysis in the 26‐week Maintenance Period of perampanel 4 mg/d or last evaluated dose (perampanel 4 or 8 mg/d) (mITT Analysis Set)

	N	Perampanel 4 mg/d		Perampanel 4 or 8 mg/d	
		n (%)	(95% CI)	n (%)	(95% CI)
Primary endpoint					
Overall seizure‐freedom rate^a^	73	46 (63.0)	(50.9‐74.0)	54 (74.0)	(62.4‐83.5)
Secondary endpoints, cumulative probability of time to:					
First seizure onset^b^	73	(30.8)	(21.5‐43.0)	(18.2)	(11.0‐29.3)
Withdrawal from the study^b^	73	(23.7)	(15.4‐35.3)	(23.3)	(15.2‐34.8)
Exploratory endpoints, seizure‐freedom rate in patients with:					
FBTCS^a^	48	31 (64.6)	(49.5‐77.8)	37 (77.1)	(62.7‐88.0)
FIAS^a^	41	24 (58.5)	(42.1‐73.7)	28 (68.3)	(51.9‐81.9)
FBTCS^a^	70	43 (61.4)	(49.0‐72.8)	51 (72.9)	(60.9‐82.8)

Abbreviations: CI, confidence interval; FBTCS, focal to bilateral tonic‐clonic seizures; FIAS, focal impaired awareness seizures; mITT, modified intent‐to‐treat.

^a^ CI was calculated using Clopper‐Pearson's exact method.

^b^ Estimated by Kaplan‐Meier method; at 26 weeks from the first date of the Maintenance Period. CI was calculated using Greenwood formula and log‐log transformation.

Results for the secondary efficacy endpoints, time to first seizure onset and time to withdrawal from the study, are presented in Table 2, and Figures S1 and S2, respectively. There were 22 (30.1%) patients who experienced seizures during the 4‐mg/d Maintenance Period. At data cutoff, the median time to first seizure onset and median time to withdrawal from the study were not estimated because <50% of patients had experienced a FOS event or discontinued from the study, respectively. However, the cumulative probability of seizure‐onset rate was 30.8% (95% CI: 21.5‐43.0), and the cumulative probability of rate of withdrawal was 23.7% (95% CI: 15.4‐35.3) at Week 26 for 4 mg/d. At the last evaluated dose (4 or 8 mg/d), the cumulative probability of seizure‐onset rate was 18.2% (95% CI: 11.0‐29.3) and of rate of withdrawal was 23.3% (95% CI: 15.2‐34.8).

In relation to the exploratory endpoint, 48 patients with FBTCS, 41 patients with FIAS, and 70 patients with FIAS and/or FBTCS entered the 4‐mg/d Maintenance Period. Seizure‐freedom rates in patients with FBTCS were achieved in 31/48 (64.6%; 95% CI: 49.5‐77.8) patients at 4 mg/d and 37/48 (77.1%; 95% CI: 62.7‐88.0) patients at the last evaluated dose (4 or 8 mg/d) (Table 2).

### 3.3 Safety outcomes

An overview of TEAEs during the 4‐mg/d or 4‐ and 8‐mg/d combined Treatment Phase is presented in Table 3. In the 4‐mg/d Treatment Phase, TEAEs occurred in 57 (64.0%) patients; 38 (42.7%) were considered treatment‐related, and all were considered mild to moderate in severity. Similarly, in the 4‐ and 8‐mg/d combined Treatment Phase, TEAEs occurred in 67 (75.3%) patients, 47 (52.8%) were considered treatment‐related, and all were considered mild to moderate. In the 4‐mg/d Treatment Phase and across the 4‐ and 8‐mg/d combined Treatment Phase, there were 9 (10.1%) patients who reported serious TEAEs, and no patients died or had TEAEs that resulted in death.

**TABLE 3:** Overview of TEAEs^a^ during the Treatment Phase (Safety Analysis Set)

	Perampanel 4 mg/d (N = 89)	Perampanel 4 and 8 mg/d (N = 89)
Any TEAE, n (%)	57 (64.0)	67 (75.3)
Treatment‐related TEAEs, n (%)^b^	38 (42.7)	47 (52.8)
Severe TEAEs, n (%)	0	0
Serious TEAEs, n (%)	9 (10.1)	9 (10.1)
Deaths	0	0
Nonfatal serious TEAEs	9 (10.1)	9 (10.1)
Life threatening	0	0
Required inpatient hospitalization or prolongation of existing hospitalization	9 (10.1)	9 (10.1)
Persistent or significant disability or incapacity	0	0
Congenital anomaly/birth defect	0	0
Important medical events	0	0
TEAEs leading to study withdrawal/perampanel dose adjustment, n (%)		
Study/perampanel withdrawal	8 (9.0)	9 (10.1)
Dose increase	1 (1.1)	1 (1.1)
Dose reduction	2 (2.2)	9 (10.1)
Dose interruption	1 (1.1)	1 (1.1)

Abbreviation: TEAE, treatment‐emergent adverse event.

^a^ For each row category, a patient with 2 or more TEAEs in that category was counted only once.

^b^ Included TEAEs considered by the investigators to be related to perampanel or TEAEs with missing causality.

The most common TEAEs (occurring in ≥2 patients) are presented in Table 4; those reported in ≥10% of patients were dizziness, nasopharyngitis, somnolence, and headache. No patients experienced TEAEs related to hostility and/or aggression.

**TABLE 4:** Most common TEAEs (occurring in ≥2 patients) during the Treatment Phase (Safety Analysis Set)

MedDRA preferred term	Perampanel 4 mg/d (N = 89)	Perampanel 4 and 8 mg/d (N = 89)
Dizziness	20 (22.5)	28 (31.5)
Nasopharyngitis	11 (12.4)	13 (14.6)
Somnolence	11 (12.4)	12 (13.5)
Headache	10 (11.2)	10 (11.2)
Epilepsy	5 (5.6)	5 (5.6)
Feeling abnormal	4 (4.5)	4 (4.5)
Blood creatine phosphokinase increased	3 (3.4)	4 (4.5)
Amnesia	2 (2.2)	2 (2.2)
Back pain	2 (2.2)	2 (2.2)
Diarrhea	2 (2.2)	2 (2.2)
Epistaxis	2 (2.2)	2 (2.2)
Irritability	2 (2.2)	3 (3.4)
Influenza	0	3 (3.4)
Weight increased	1 (1.1)	2 (2.2)
Hypersomnia	1 (1.1)	2 (2.2)
Anxiety	0	2 (2.2)

Abbreviations: MedDRA, Medical Dictionary for Regulatory Activities; TEAE, treatment‐emergent adverse event.

There were no clinically important changes in laboratory parameters, vital signs, or weight at the end of treatment. Shift analyses revealed no changes of clinical concern for urinalysis parameters and ECG results.

## 4 DISCUSSION

The aims of this study were to investigate the efficacy and safety of perampanel monotherapy in currently untreated patients with FOS. This is the first study to investigate perampanel administered as monotherapy to patients with newly diagnosed epilepsy. Perampanel 4‐mg/d monotherapy, the lowest dosage administered in this study, was associated with efficacy for up to 26 weeks in primarily newly diagnosed patients with FOS (with or without FBTCS) from Japan and South Korea. For the primary endpoint (seizure‐freedom rate for the mITT Analysis Set [at 4 mg/d]: 63.0% [95% CI: 50.9‐74.0]) and based on other studies of AEDs as monotherapy,^15^, ^16^, ^17^, ^18^, ^19^ prespecified efficacy criteria were fulfilled as the lower 95% CI of the seizure‐freedom rate during the 26‐week Maintenance Period was above the threshold of 40%. Efficacy endpoints were assessed in the mITT Analysis Set, after the Titration Period, since the apparent terminal half‐life of perampanel is ~105 hours, with time to reach steady state considered to be 10‐19 days.^20^ The seizure‐freedom rate in the sensitivity analysis for the ITT Analysis Set at 4 mg/d (51.7%; 95% CI: 40.8‐62.4) was also above this threshold. Seizure‐freedom rates appeared broadly consistent regardless of FOS type. Furthermore, 8 patients achieved seizure freedom by increasing the dose and seizure‐freedom rates at the last evaluated dose (4 or 8 mg/d) were 74.0% (95% CI: 62.4‐83.5) for the overall mITT Analysis Set.

For topiramate, a double‐blind, randomized dose‐controlled study showed that at 6 months, the seizure‐freedom rate was 83% in the 400‐mg/d group and 71% in the 50‐mg/d group.^16^ However, all ITT patients had only 1 or 2 seizures during the 3‐month baseline period and the median pretreatment seizure frequency per month was 0.33. The higher seizure‐freedom rate in the study by Arroyo et al could have been due to the lower pretreatment seizure frequency than in our study. For 4 widely used AEDs (phenobarbitone, phenytoin, carbamazepine, and sodium valproate), an open‐label, randomized study showed that the overall seizure‐freedom rate was 51% at 6 months.^18^ For levetiracetam, an open‐label, randomized study showed that the 6‐month seizure‐freedom rate with levetiracetam monotherapy in the response‐based titration group (1000‐2000 mg/d) was 45/61 (73.8%) in patients with FOS.^19^ Also, in a similarly designed, open‐label study that assessed the efficacy and safety of lamotrigine monotherapy (100‐400 mg/d) in patients with newly diagnosed or recurrent epilepsy in Japan and South Korea, the seizure‐freedom rate was 28/65 (43.1%) patients across all seizure types, and 22/55 (40.0%) patients for FOS specifically.^21^ Overall, the seizure‐freedom rate of 63.0% at perampanel 4 mg/d in our study for the mITT Analysis Set is comparable to other AEDs in other monotherapy trials in patients with FOS.

Our study was performed in order to determine the efficacy of perampanel 4 mg/d as monotherapy; patients were only up‐titrated to 8 mg/d if they experienced a seizure at the lower 4‐mg/d dose. The perampanel monotherapy dose of 4 mg/d was at the lowest end of the effective perampanel 4‐12 mg/d dose range previously explored across the double‐blind, placebo‐controlled, randomized Phase III studies of adjunctive perampanel involving patients with pharmacoresistant FOS.^22^, ^23^, ^24^, ^25^ Perampanel 4 mg/d as adjunctive therapy showed efficacy in patients with inadequately controlled FOS in Study 306 (NCT00700310), but failed to show efficacy in the Asia‐Pacific Study 335 (NCT01618695).^24^, ^25^ This difference may have been due to a more drug‐refractory patient population in Study 335 compared with Study 306,^24^ and the fact that a higher proportion of these patients were receiving enzyme‐inducing AEDs, which would have resulted in reduced systemic exposure to perampanel.^26^ This is in keeping with the most comprehensive model‐predicted population pharmacokinetic/pharmacodynamic analyses of adjunctive perampanel performed to date. These analyses explored the relationship between perampanel exposure and 28‐day average seizure frequency and responder probability, and showed that while concomitant use of enzyme‐inducing AEDs may require administration of a higher perampanel dose, there was no effect of a variety of intrinsic factors including the Japanese or the Chinese race.^27^, ^28^

As noted above, and comparing our result with previous studies, the efficacy of perampanel 4 mg/d seems comparable to other AEDs administered as monotherapy. Other AEDs, for example, levetiracetam, lacosamide, and lamotrigine, are approved as adjunctive therapy and monotherapy by the FDA.^29^, ^30^, ^31^ In the United States, the maintenance dose for levetiracetam is 1000–3000 mg/d for both adjunctive therapy and monotherapy. For lacosamide, the maintenance dose for adjunctive therapy is 200‐400 mg/d, and 300‐400 mg/d for monotherapy.^30^ For lamotrigine, the maintenance dose for adjunctive therapy is 225‐375 mg/d (in patients not taking carbamazepine, phenytoin, phenobarbital, primidone, or valproate), and 500 mg/d for conversion from adjunctive therapy to monotherapy.^31^ In the previously mentioned study of levetiracetam monotherapy, the 6‐month seizure‐freedom rate was 45/61 (73.8%) patients in the 1000‐2000 mg/d response‐based titration group and 2/9 (22.2%) patients in the 3000 mg/d forced titration group.^19^ In a larger prospective, noninferiority, double‐blind monotherapy trial comparing levetiracetam and controlled‐release carbamazepine in newly diagnosed epilepsy, the seizure‐freedom rate was 66.7% (190/285) for patients receiving levetiracetam 1000‐3000 mg/d in the ITT Analysis Set.^32^ For levetiracetam, 80.1% of patients received the lowest effective dose (1000 mg/d) with the remaining patients receiving 2000 or 3000 mg/d. Perampanel monotherapy administered as 4 to 8 mg/d showed comparable efficacy in terms of the seizure‐freedom rates to those previously reported for levetiracetam.^19^, ^32^

When perampanel was administered as monotherapy at doses of 4 to 8 mg/d, treatment was well tolerated and no new safety signals were identified in our study. The withdrawal rate due to TEAEs was low: of the 57 (64.0%) patients who reported at least 1 TEAE during the 4‐mg/d Treatment Phase, only 8 (9.0%) patients withdrew due to TEAEs. The safety profile was consistent with that reported for perampanel 4 mg/d in the Phase III studies of adjunctive perampanel (Studies 306 and 335).^24^, ^25^ However, it is important to note that the Phase III studies of adjunctive perampanel were conducted in patients with refractory epilepsy, whereas Study 342 was conducted in patients with epilepsy, almost all (94.4%) of whom were newly diagnosed. Despite this difference, the TEAE profiles were consistent as the most common TEAEs in Study 342 at 4 mg/d included dizziness (n = 20; 22.5%), nasopharyngitis and somnolence (each n = 11; 12.4%), headache (n = 10; 11.2%), and epilepsy (n = 5; 5.6%), while the most common TEAEs in Studies 306 and 335 included dizziness, somnolence, headache, nasopharyngitis, and fatigue.^24^, ^25^ In addition, TEAE rates were generally similar across the studies for the perampanel 4‐mg/d dose (Study 342, 64.0%; Study 306, 64.5%; and Study 335, 68.8%).^24^, ^25^

There are potential limitations that should be considered during interpretation of this study. Firstly, this was an open‐label study without a control arm due to ethical concerns, as inclusion of placebo groups in AED monotherapy studies may prevent patients from receiving care that may be critical for epilepsy management.^14^ This study focused on the efficacy of perampanel 4 mg/d during the Maintenance Period only, with supporting data from the last evaluated dose (4 or 8 mg/d) if the patient experienced a seizure while receiving 4 mg/d; further data from the Extension Phase are yet to be reported. These data will be instrumental for assessing long‐term seizure‐freedom rates. In addition, prospective studies are needed to assess the efficacy of perampanel as monotherapy analyzed according to the number of previously received AEDs or seizure types.

In conclusion, the results from this open‐label study demonstrated that perampanel monotherapy (4 to 8 mg/d) may be an efficacious and well‐tolerated treatment option in currently untreated patients aged ≥12 years with different types of FOS.

## CONFLICT OF INTEREST

Takamichi Yamamoto has received speaker's honoraria from Daiichi‐Sankyo, Eisai, Otsuka Pharmaceutical, and UCB Pharma, and has participated in advisory boards for Eisai. Hirotomo Ninomiya has received honoraria for lectures from Daiichi‐Sankyo, Eisai, Otsuka Pharmaceutical, and UCB Pharma. Yuichi Kubota has received honoraria from Daiichi‐Sankyo, Eisai, Otsuka Pharmaceutical, and UCB Pharma, and has participated in advisory boards for Eisai. Tohru Hoshida has received honoraria from Daiichi‐Sankyo and Eisai. Koji Iida has received honoraria and research grants from Daiichi‐Sankyo, Eisai, Otsuka Pharmaceutical, and UCB Pharma. Taku Ochiai has received honoraria from Daiichi‐Sankyo, Eisai, Otsuka Pharmaceutical, and UCB Pharma. Sung Chul Lim, Won Chul Shin, Dong Wook Kim, Dong Jin Shin, and Ji Hyun Kim have no real or apparent conflicts of interest to disclose. Risa Matsunaga and Hidetaka Hiramatsu are employees of Eisai Co., Ltd. Hiroyuki Higashiyama is a former employee of Eisai Co., Ltd. We confirm that we have read the Journal's position on issues involved in ethical publication and affirm that this report is consistent with those guidelines.

## AUTHOR CONTRIBUTIONS

Takamichi Yamamoto is a medical adviser of this study. He contributed to the study design and protocol development. Risa Matsunaga, Hiroyuki Higashiyama, and Hidetaka Hiramatsu contributed to the study design, protocol development, and data analysis (funded by Eisai Co., Ltd.). Sung Chul Lim, Hirotomo Ninomiya, Yuichi Kubota, Won Chul Shin, Dong Wook Kim, Dong Jin Shin, Tohru Hoshida, Koji Iida, Taku Ochiai, and Ji Hyun Kim coordinated recruitment of patients into the study in their respective departments. Takamichi Yamamoto, Risa Matsunaga, Hiroyuki Higashiyama, and Hidetaka Hiramatsu contributed to the preparation of the manuscript. All authors had access to the study data, were involved in the decision to submit this article for publication, contributed to data interpretation, reviewed the manuscript, and approved the final version.

## Supporting information

Fig S1

Fig S2
