# Supplementary figures and images for: Efficacy and safety of perampanel monotherapy in patients with focal‐onset seizures with newly diagnosed epilepsy or recurrence of epilepsy after a period of remission: The open‐label Study 342 (FREEDOM Study)
Source: Epilepsia Open. 2020 Jun 7;5(2):274–84. doi: 10.1002/epi4.12398 (PMC7278556; doi:10.1002/epi4.12398)

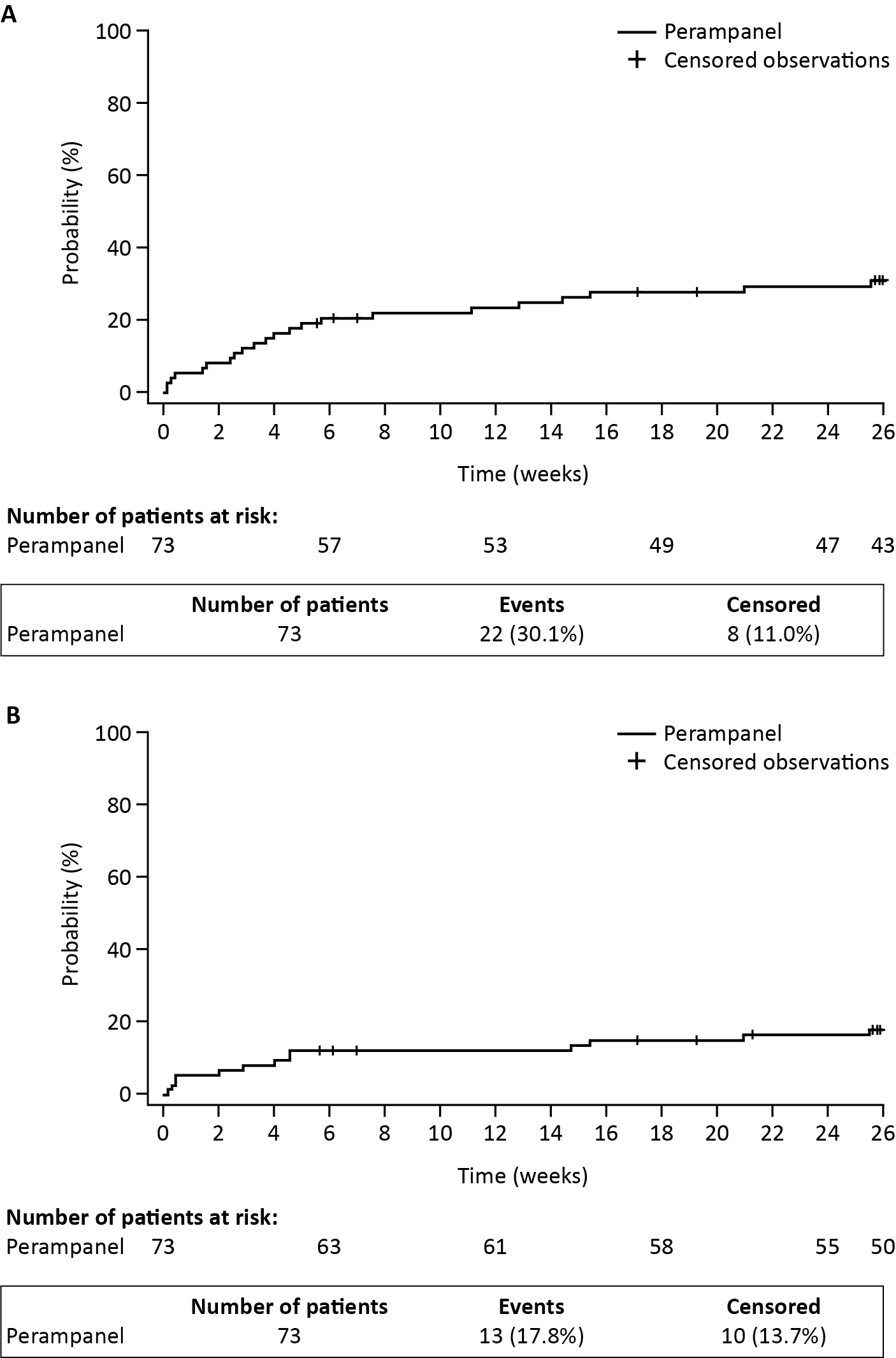

Supplement: Supplementary file 1 — Fig S1 [file EPI4-5-274-s001.tif]

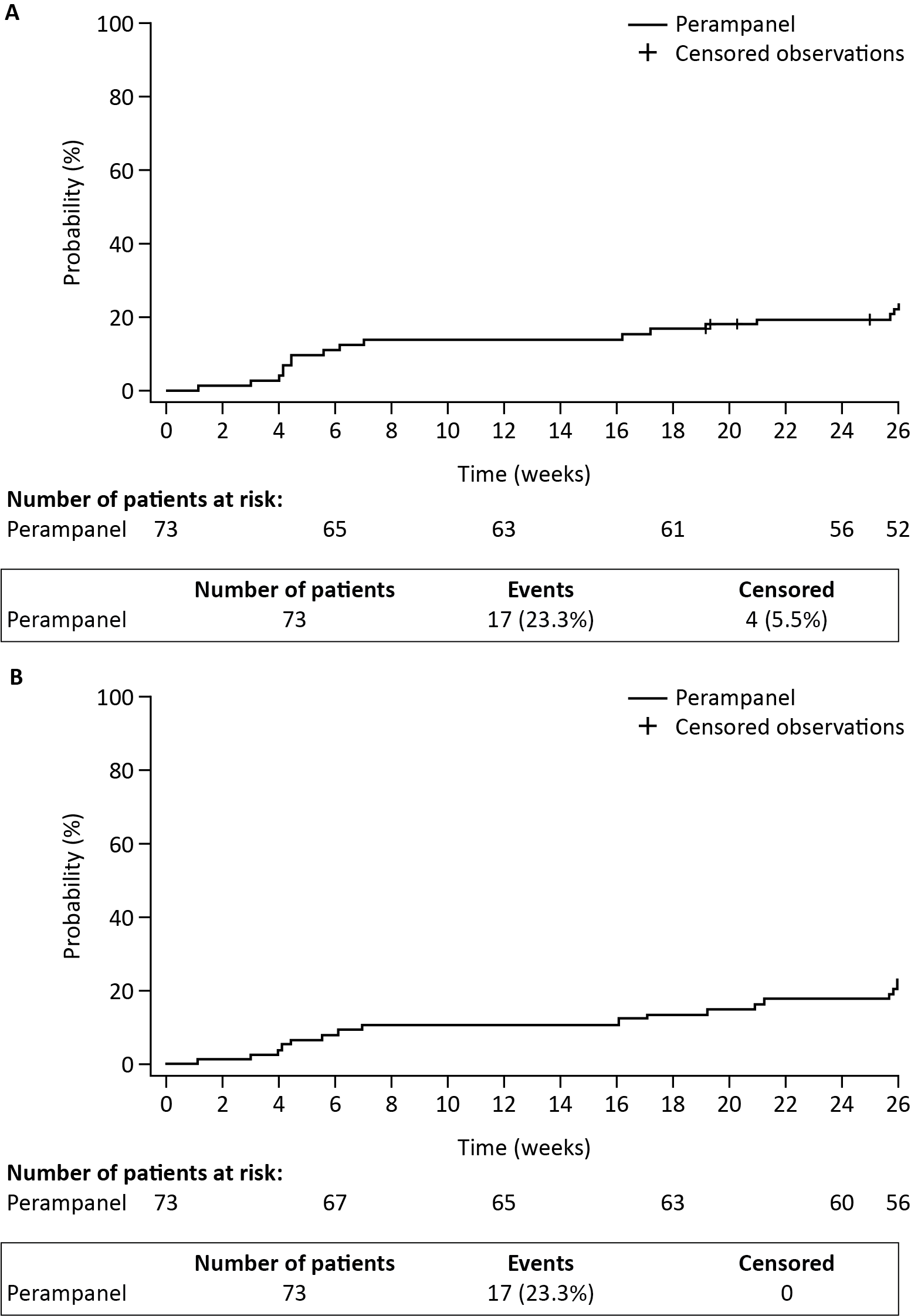

Supplement: Supplementary file 2 — Fig S2 [file EPI4-5-274-s002.tif]
